# FDA-approved antisense oligonucleotide therapies for duchenne muscular dystrophy: current status and future outlook

**DOI:** 10.1080/15476286.2026.2689117

**Published:** 2026-06-21

**Authors:** Hidenori Moriyama, Shiori Moriyama, Toshifumi Yokota

**Affiliations:** a Department of Medical Genetics, Faculty of Medicine and Dentistry, University of Alberta, Edmonton, Canada; b Department of Neurology, Tokyo Metropolitan Children’s Medical Center, Tokyo, Japan

**Keywords:** Duchenne muscular dystrophy, dystrophin, exon skipping, antisense oligonucleotide, FDA, eteplirsen, golodirsen, viltolarsen, casimersen

## Abstract

Duchenne muscular dystrophy (DMD) is a fatal X-linked recessive disorder caused by dystrophin deficiency. Antisense oligonucleotide (ASO)-mediated exon skipping has emerged as a cornerstone of DMD therapy to restore dystrophin expression. This review provides a comprehensive overview of the four FDA-approved ASO therapies – eteplirsen, golodirsen, viltolarsen, and casimersen – tracing their journey from pivotal clinical trials to post-marketing updates. While the development and clinical evaluation of these agents have established a pioneering framework for rare genetic diseases, they have also highlighted critical challenges. These include complexities in clinical trial design, discrepancies between preclinical efficacy and clinical outcomes, real-world burdens, and limited patient eligibility. Furthermore, the FDA’s accelerated approval of these therapies based on limited clinical data remains a subject of ongoing debate. Confirmatory trials to verify clinical efficacy and long-term follow-up studies are actively underway. Concurrently, intensive research is focused on developing next-generation ASOs to achieve enhanced therapeutic efficacy and definitive clinical outcomes. Elucidating the trajectory of research and development in this field offers profound insights for shaping future therapeutic strategies in rare diseases.

## 1. Introduction

Duchenne muscular dystrophy (DMD) is the most prevalent form of muscular dystrophy, a group of genetic muscle diseases characterized primarily by the necrosis and regeneration of skeletal muscle. Global epidemiological studies report a DMD prevalence of 7.1 cases per 100,000 males (95% confidence interval [CI]: 5.0–10.1) and an incidence at birth of 19.8 cases per 100,000 live male births (95% CI: 16.6–23.6) [1]. The causative gene for this condition is the *DMD* gene located at Xp21, following an X-linked recessive inheritance pattern. Spanning a massive genomic region of approximately 2.2 Mb and consisting of 79 exons, the *DMD* gene locus generates multiple transcripts and protein isoforms via alternative promoters. Among these, the full-length major skeletal muscle isoform (molecular weight 427 kDa; 3,685 amino acids) plays a pivotal role in the structural stabilization of the sarcolemma by linking the cytoskeleton to the extracellular matrix via the dystrophin-associated protein complex [2]. Approximately 61% of genetic mutations in DMD are exon deletions and 9% are duplications, with the remainder composed of small mutations such as microdeletions, nonsense mutations, and splice-site mutations [3].

The severity of clinical symptoms is heavily dependent on the ‘reading frame rule’. Out-of-frame mutations, which disrupt the translational reading frame, result in a complete deficiency of functional dystrophin and lead to the severe DMD phenotype. In contrast, in-frame mutations maintain the reading frame and allow for the production of internally deleted but partially functional dystrophin, resulting in the clinically milder Becker muscular dystrophy (BMD). This rule holds true for 93% of deletion mutations and 66% of duplication mutations, serving as the theoretical foundation for molecular therapeutic strategies using nucleic acid drugs [3]. The absence of dystrophin leads to membrane fragility in muscle fibres, triggering an ongoing cycle of necrosis and regeneration that ultimately results in the replacement of muscle tissue with fat and fibrous tissue [4]. Consequently, patients present with motor dysfunction between ages 3 and 5, lose the ability to ambulate by ages 10 to 12, and require essential management for respiratory failure and cardiomyopathy from adolescence onward.

For many years, DMD treatment focused on symptomatic management and complication care. Advances in multidisciplinary neuromuscular care, including proactive cardiac management, corticosteroid therapy, respiratory support, scoliosis management, nutritional optimization, and non-invasive ventilation, together with the establishment of standardized care guidelines, have improved survival and quality of life in patients with DMD. A recent natural history study in the UK reported a median life expectancy of 25.64 years, extending to 26.47 years for those born after 1990 [5]. Meta-analyses indicate that the introduction of ventilation therapy has significantly contributed to improved prognosis, with median survival increasing from 19.0 years in untreated groups to 29.9 years in treated groups [6]. While long-term corticosteroid therapy helps prolong ambulation and preserve cardiopulmonary function, managing its associated adverse effects remains a major clinical challenge. These long-term side effects include obesity, growth suppression, osteoporosis, vertebral fractures, cataracts, and behavioural disturbances [7]. In response, Vamorolone, a dissociative steroid designed to reduce side effects, and Givinostat, a histone deacetylase (HDAC) inhibitor expected to delay disease progression when used alongside steroids, were both approved by the U.S. Food and Drug Administration (FDA) between 2023 and 2024 (Table 1) [8,9].

**Table 1.** FDA-approved drugs for DMD.

Drug name (Brand)	Mechanism of action	Indication and key consideration	Method of administration	Company	Approval year
Eteplirsen (Exondys 51)	Exon 51 Skipping	Patients with mutations amenable to Exon 51 skipping	Weekly IV infusion	Sarepta Therapeutics	2016
Golodirsen (Vyondys 53)	Exon 53 Skipping	Patients with mutations amenable to Exon 53 skipping	Weekly IV infusion	Sarepta Therapeutics	2019
Viltolarsen (Viltepso)	Exon 53 Skipping	Patients with mutations amenable to Exon 53 skipping	Weekly IV infusion	NS Pharma	2020
Casimersen (Amondys 45)	Exon 45 Skipping	Patients with mutations amenable to Exon 45 skipping	Weekly IV infusion	Sarepta Therapeutics	2021
Delandistrogene moxeparvovec-rokl (Elevidys)	Gene therapy (microdystrophin)	4 years and older Contraindications:patients with deletions of exon 8 or 9, and patients who are positive for anti-AAVrh74 antibodies.	Single-dose IV infusion	Sarepta Therapeutics	2023
Deflazacort (Emflaza)	Corticosteroid	2 years and older	Oral	PTC Therapeutics	2017
Vamorolone (Agamree)	Dissociative Steroid	2 years and older	Oral	Santhera Pharmaceuticals	2023
Givinostat (Duvyzat)	HDAC Inhibitor	6 years and older	Oral	Italfarmaco	2024

A list of FDA-approved drugs for DMD as of May 2026. IV, intravenous; HDAC, Histone deacetylase.

**Figure 1.**
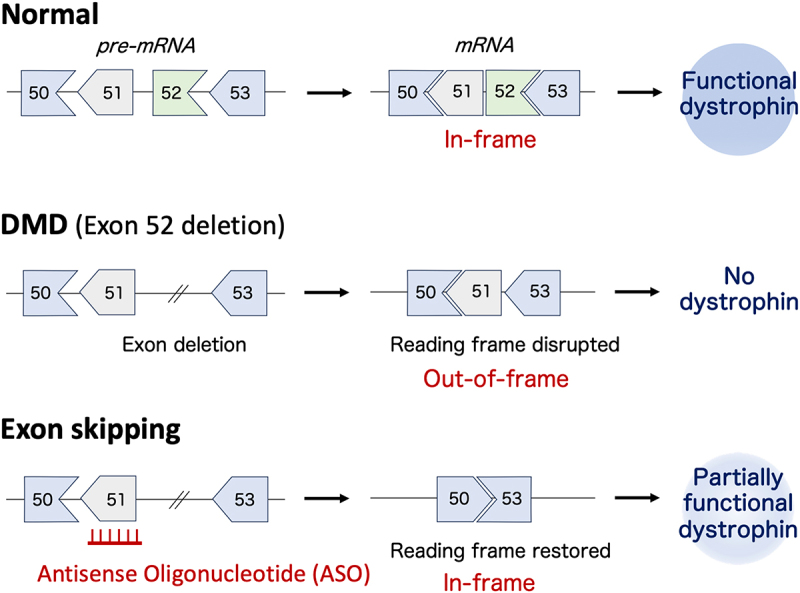
Mechanism of exon skipping therapy using ASO for DMD. In the normal state, all exons of the *dystrophin* gene are spliced together in-frame to produce a functional dystrophin protein (top). As a specific example of DMD, the deletion of exon 52 disrupts the reading frame, resulting in an out-of-frame mRNA sequence between exons 51 and 53 that prevents protein production (middle). To treat this, an ASO is used to induce exon 51 skipping, intentionally removing the additional exon to restore the reading frame between exons 50 and 53. This therapeutic skipping of exon 51 in the presence of an exon 52 deletion allows for the synthesis of an internally-deleted but partially functional dystrophin protein (bottom).

Parallel to these supportive therapies, the development of more fundamental molecular treatments aimed at targeting *DMD* gene mutations or restoring functional dystrophin has made dramatic progress. Currently, the core of these advancements lies in exon skipping therapy using antisense oligonucleotides (ASOs) and gene replacement therapy (Table 1). Approved by the FDA in 2023, Delandistrogene moxeparvovec-rokl (Elevidys) is a gene replacement therapy that uses an adeno-associated virus (AAV) vector to deliver an internally-deleted *DMD* gene into muscle cells. Since the full length of the *DMD* gene exceeds the packaging capacity of AAV, a ‘micro-dystrophin’ retaining essential functional domains is utilized. While this treatment offers the potential for long-term dystrophin expression following a single dose and is applicable to a wide range of mutations with some exceptions, its clinical efficacy remains a subject of ongoing debate, and challenges regarding immune responses and expression durability persist [10,11]. Meanwhile, ASOs are short, single-stranded nucleic acids that bind complementarily to target RNA to regulate gene expression through mechanisms such as RNA degradation, splicing modification, or translational inhibition [12,13]. Among these, the exon skipping strategy using ASO in DMD involves intentionally excluding a target exon during the splicing process to restore the disrupted reading frame, thereby converting the severe DMD phenotype into a milder BMD phenotype expressing an internally-deleted dystrophin (Figure 1) [14]. Notably, mutation hotspot regions exist within the *DMD* gene, with the largest clustering between exons 45 and 55. Because exon skipping therapy is mutation-specific, development has prioritized this region to cover a larger patient population. To date, four ASO drugs targeting exons 45, 51, or 53 skipping have received FDA approval; however, these therapies collectively apply to approximately 27% of all DMD patients [15]. All of these approved drugs utilize a phosphorodiamidate morpholino oligomer (PMO) chemistry, known for its superior chemical stability and safety profile.

As the landscape of DMD treatment undergoes a major paradigm shift, this review focuses specifically on the four FDA-approved exon-skipping therapies. We comprehensively outline their developmental histories, clinical trial designs and outcomes, and trends following FDA approval. Establishing proof-of-concept for dystrophin restoration via ASO-mediated exon skipping, which led to FDA approvals, has brought newfound hope to eligible DMD patients, their families, and individuals suffering from other intractable genetic diseases. However, these accelerated approvals, primarily based on the biomarker of modest increases in dystrophin levels, have sparked significant debate, and discussions regarding their definitive clinical benefit remain ongoing. Furthermore, challenges such as prohibitive costs and limited patient eligibility persist. The cumulative research and development in this space have highlighted current limitations and unmet medical needs, thereby serving as a valuable platform for the engineering of next-generation ASOs. Tracing the trajectory of ASO development in DMD will undoubtedly provide critical insights into the advancement of nucleic acid therapeutics for other rare conditions, as well as the design of next-generation modalities for various intractable diseases.

## 2. Eteplirsen (Exondys 51)

Eteplirsen developed by Sarepta Therapeutics was the first exon-skipping ASO to receive FDA approval in 2016. It is designed to hybridize to and induce the skipping of exon 51. This agent is applicable to approximately 17.2–20.5% of DMD patients with deletion mutations, representing 10.6–14% of the total DMD population – the largest single subgroup eligible for exon skipping [15–17]. AVI-4658 (eteplirsen) was selected based on demonstrated exon 51 skipping efficiency in human muscle cells and transgenic mice harbouring the entire human *DMD* locus [18]. Table 2 summarizes the main clinical trials related to eteplirsen.

**Table 2.** Major clinical trials of eteplirsen.

NCT Number (Study Name)	Phase	Study Overview	Patient Profile, Dosing Scheme	Key Outcomes
NCT00844597	1/2	Open-label, dose-escalation study to assess the safety and tolerability of eteplirsen	19 boys with DMD (ages 5–15) received 0.5, 1, 2, 4, 10, or 20 mg/kg IV once weekly for 12 weeks.	No drug-related serious adverse events. Dose-dependent expression of dystrophin protein.
NCT01396239 (4658-us-201)	2	Randomized, double-blind, placebo-controlled study to assess the efficacy, safety, tolerability, and pharmacokinetics of eteplirsen	12 boys with DMD (ages 7–13) received 30, 50 mg/kg or placebo IV once weekly for 24 weeks.	At 48 weeks, increase in dystrophin-positive fibres and 6MWD compared to the placebo group. At 180 weeks, dystrophin expression was 0.93%.
NCT01540409 (4658-us-202)	2	Open-label extension of NCT01396239	12 patients who completed NCT01396239 received additional IV 30 or 50 mg/kg IV once weekly for 212 weeks.	
NCT02255552 (PROMOVI)	3	Non-randomized, open-label study to evaluate the efficacy and safety of eteplirsen	79 boys with DMD (ages 7–16) received 30 mg/kg IV once weekly for 96 weeks.	At 96 weeks, increase in exon skipping (1.091%) and dystrophin expression (0.63%) from baseline. Reduced decline in 6MWD and respiratory function compared to untreated external cohort.
NCT06606340 (EVOLVE, 4658–403)	4	Long-term observational study of eteplirsen	30 mg/kg IV once weekly (target enrolment: 300 patients).	Study completion expected in 2033.
NCT03992430 (MIS51ON)	3	Randomized, double-Blind study to evaluate the safety and efficacy of high doses of eteplirsen	160 boys with DMD (ages 4–13) receiving 30, 100, or 200 mg/kg IV once weekly for 144 weeks.	Not recruiting. Study completion expected in October 2026.

Information based on ClinicalTrials.gov as of May 2026. DMD, Duchenne muscular dystrophy; IV, intravenous; 6MWD, 6-minute walk distance.

NCT00159250 was the earliest phase 1/2, single-blind, placebo-controlled, dose-escalation clinical trial and served as a proof-of-concept study [19]. This study evaluated the safety of intramuscular eteplirsen injection and verified whether it could induce exon 51 skipping and restore dystrophin protein expression. Seven DMD patients received intramuscular injections of 0.09 mg or 0.9 mg of eteplirsen into the extensor digitorum brevis muscle, with saline injected into the contralateral muscle as a control. No serious adverse events were reported. Immunohistochemical analysis for muscle biopsy samples obtained 3–4 weeks post-administration demonstrated an increase in dystrophin expression in the high-dose group, with the mean staining intensity ranging from 22% to 32% of that in healthy control muscles. Although Western blot analysis was not quantified in this study, it confirmed the restoration of dystrophin protein expression in the high-dose group, thereby validating the feasibility of the therapeutic concept.

NCT00844597 followed as an open-label, phase 1/2, dose-escalation study [20]. Nineteen ambulatory DMD boys aged 5–15 received weekly intravenous infusions of eteplirsen (0.5–20 mg/kg) for 12 weeks. The drug was well-tolerated, with no serious drug-related adverse events observed. Exon 51 skipping was confirmed across all dosage groups; notably, those receiving 2 mg/kg or more showed dose-dependent dystrophin restoration. Additionally, a reduction in cytotoxic T cells within the muscle tissue was observed in the high-dose groups. These results suggested that eteplirsen could safely and effectively restore dystrophin and potentially modify the disease course of DMD.

NCT01396239 (Study 4658-US-201) was a phase 2, double-blind, placebo-controlled trial that evaluated the efficacy and safety of eteplirsen [21]. Twelve ambulatory boys with DMD (aged 7–13) were randomized to receive weekly intravenous doses of 30 mg/kg or 50 mg/kg of eteplirsen, or a placebo, for 24 weeks. From week 25 onwards, patients in the placebo group were transitioned to eteplirsen (30 or 50 mg/kg) in an open-label extension study (NCT01540409, Study 4658-US-202) [21,22]. At week 48, the eteplirsen-treated groups showed a significant increase in the percentage of dystrophin-positive fibres (52% of normal levels in the 30 mg/kg group; 43% in the 50 mg/kg group). Furthermore, the eteplirsen groups demonstrated a significant maintenance effect in the six-minute walk test (6MWT), outperforming the placebo (delayed-treatment) group by 67.3 metres, though two participants in the treatment group who became non-ambulatory were excluded from this analysis. By 36 months, the eteplirsen-treated group maintained significantly better ambulatory capacity – with a 151-metre difference in 6MWT distance – and showed a lower rate of loss of ambulation compared to an external control group of baseline-matched patients from natural history data in Belgium and Italy [22]. Respiratory function remained stable, and the drug was well-tolerated. It should be noted that a re-verification via Western blot requested during the FDA approval process, using muscle biopsy samples collected at week 180, reported the mean dystrophin level to be 0.93% of normal [23].

NCT02255552 (PROMOVI trial) was a larger open-label phase 3 clinical trial [24]. Seventy-nine ambulatory boys with DMD (aged 7–16) received weekly intravenous doses of 30 mg/kg eteplirsen for 96 weeks. At the 96-week mark, the exon skipping rate increased 18.7-fold from baseline (1.091%), and dystrophin expression increased 7-fold (0.63% by Western blot). Although the trial initially enrolled patients ineligible for exon 51 skipping as a control group, direct comparison was deemed inappropriate due to significant genotype-driven differences in clinical progression. Instead, functional outcomes were compared through post hoc analyses using external controls. The results revealed that, compared to natural history data for exon 51-eligible patients, those treated with eteplirsen experienced a suppressed decline in 6MWT distance and respiratory function.

In 2016, the FDA granted accelerated approval to eteplirsen based on data from NCT01396239 and NCT01540409, as well as preliminary data from NCT02255552. However, this approval process was the subject of intense debate due to several factors [23,25–27]. The trials were constrained by an extremely small sample size and the open-label design of the latter half of the phase 2 and the phase 3 trials. The magnitude of dystrophin restoration was extremely limited; western blot quantification revealed that dystrophin levels remained below 1% after 48 to 180 weeks of treatment. This modest increase led some FDA reviewers to argue that such small amounts were negligible and unlikely to provide any therapeutic benefit [26]. Furthermore, the interpretation of functional efficacy was highly problematic. In the 6MWT analyses, two treated patients who lost ambulation within three months of trial initiation were effectively excluded from meaningful long-term longitudinal comparisons. Because the trial lacked a concurrent placebo control group beyond 24 weeks, the treated cohort could only be evaluated against external historical controls selected from natural history data [22]. FDA reviewers remained unconvinced that these small-group external comparisons demonstrated any real clinical benefit. Despite significant internal disagreement within the FDA, the regulatory decision ultimately resulted in accelerated approval.

Patients from the NCT01396239/NCT01540409 studies have continued eteplirsen treatment, and long-term real-world clinical data have been reported through comparisons with external, untreated historical control cohorts. Long-term follow-up (3–7 years) of these patients indicates a stabilized rate of respiratory decline and a prolonged period of ambulation [28–32]. Furthermore, reports evaluating survival impact over up to 8 years of continuous treatment showed a significant reduction in mortality compared specifically to an untreated external cohort, with a median survival extension of 5.4 years [33]. Consequently, evidence regarding the long-term clinical benefit of continuous eteplirsen administration is accumulating, albeit largely through comparisons with external untreated cohorts.

As part of post-marketing long-term observation, the phase 4 clinical trial NCT06606340 (EVOLVE) is currently underway, with a planned follow-up of up to 5 years [34]. Additionally, NCT03992430 (MIS51ON) is an ongoing phase 3 trial investigating the safety and efficacy of high-dose eteplirsen [35]. This study compares the therapeutic effects and safety of 100 mg/kg or 200 mg/kg doses against the standard 30 mg/kg dose. Patient enrolment is complete, and the results are highly anticipated, as they are expected to significantly influence future ASO dosing strategies.

During clinical trials and regulatory review processes, the difficulty of quantifying trace amounts of restored dystrophin with high precision became a glaring challenge. In addition to variations depending on the muscle biopsy site (sampling error), conventional Western blotting and immunostaining methods exhibited high inter-operator and inter-facility data variability, as well as low reproducibility. Currently, progress is being made towards using standardized reporting methods via Western blotting to enhance objectivity, alongside the increasing adoption of capillary Western blotting and mass spectrometry [36–38]. Regarding the further improvement of efficacy, research suggesting that alternative sequence selection could potentially enhance effectiveness underscores the importance of molecular optimization, which is increasingly expected to leverage in silico tools for more precise sequence prediction in future therapies [39].

## 3. Golodirsen (Vyondys 53)

Golodirsen developed by Sarepta Therapeutics is an ASO designed to induce the skipping of exon 53, receiving FDA approval in 2019 [40]. This agent is applicable to approximately 11.8–13.7% of DMD patients with deletion mutations, representing 8.1–8.3% of the total DMD population [15,16]. Table 3 summarizes the main clinical trials related to golodirsen.

**Table 3.** Major clinical trials of golodirsen.

NCT number (Study name)	Phase	Study overview	Patient profile, dosing scheme	Key outcomes
NCT02310906 (SKIP-NMD, 4053–101)	1/2	Randomized, double-blind, placebo-controlled study to assess the safety, tolerability and pharmacokinetics of golodirsen	12 boys with DMD (ages 6–15) received golodirsen (escalating dose of 4, 10, 20, 30 mg/kg) or placebo IV once weekly for 12 weeks.	At 48 weeks, increase in exon skipping (18.95%) and dystrophin expression (1.02%) from baseline. Reduced decline in 6MWD and respiratory function at 3 years compared to untreated cohort.
NCT03532542 (4045–302)	3	Open-label extension of NCT02310906	25 DMD patients, including the 12 who completed NCT02310906, received 30 mg/kg IV once weekly for 168 weeks.	
NCT02500381 (ESSENCE, 4045–301)	3	Randomized, double-blind, placebo-controlled study to evaluate the efficacy and safety of golodirsen (and casimersen)	228 boys with DMD (ages 6–13) received 30 mg/kg or placebo IV once weekly for 96 weeks. All participants received active treatment for 48 weeks in the subsequent open-label extension.	At 96 weeks, the change from baseline in the 4-step ascend velocity did not reach statistical significance.
NCT06606340 (EVOLVE, 4658–403)	4	Long-term observational study of golodirsen	30 mg/kg IV once weekly (target enrolment: 300 patients).	Study completion expected in 2033.

Information based on ClinicalTrials.gov and announcement from the company as of May 2026. DMD, Duchenne muscular dystrophy; IV, intravenous; 6MWD, 6-minute walk distance.

The initial clinical evaluation was conducted through NCT02310906 (SKIP-NMD), a randomized, double-blind, placebo-controlled phase 1/2 trial, and its subsequent open-label extension (NCT03532542). In part 1, twelve DMD boys aged 6–15 were randomized (8 to SRP-4053 [golodirsen] and 4 to placebo) to receive weekly intravenous infusions for 12 weeks with dose escalation (4, 10, 20, and 30 mg/kg). This phase evaluated safety, tolerability, pharmacokinetics, and dystrophin expression [41,42]. In part 2, an open-label extension included the original 12 participants plus 13 additional patients (total *n* = 25), all receiving 30 mg/kg of golodirsen weekly for up to 168 weeks. The drug was well-tolerated, with a short plasma half-life of 3–6 hours even at the 30 mg/kg dose. By week 48, the mean level of exon skipping reached 18.95%, while the mean dystrophin level measured by Western blot reached 1.019% of normal, representing an approximate 16-fold rise from baseline [41]. Supplemental analysis of these samples revealed that even low levels of dystrophin restoration led to the recovery of dystrophin-associated proteins involved in sarcolemmal stability (e.g. α-sarcoglycan and β-dystroglycan) and the suppression of pathological muscle regeneration [43].

Despite a lack of definitive data confirming clinical functional improvement at the time, the FDA granted accelerated approval to golodirsen in 2019, primarily based on the biomarker data showing increased dystrophin [44]. Long-term follow-up reports at the three-year mark from baseline – comparing treated patients to external natural history data matched for age and corticosteroid use – indicated that the treated group experienced a slower decline in 6MWT distance, a lower rate of loss of ambulation, and better maintenance of respiratory function. Most adverse events were mild and deemed unrelated to the study drug, with no reports of treatment discontinuation or death due to safety concerns [42].

A larger phase 3, double-blind, randomized, placebo-controlled trial, NCT02500381 (ESSENCE), was conducted as a confirmatory study to verify the clinical benefit required by the FDA, enrolling a total of 228 patients across both the golodirsen and casimersen cohorts [45]. Participants were randomized 2:1 to receive either the active drug or placebo for 96 weeks, followed by an open-label extension up to week 144. This trial evaluated clinical efficacy through physical function tests (including the 6MWT), muscle biopsies, and safety assessments. Although the trial concluded in November 2025, it ultimately failed to meet its primary clinical endpoint; Sarepta Therapeutics announced top-line results, stating that the change from baseline in the 4-Step ascend velocity at week 96 did not reach statistical significance [46]. The company noted, however, that the trial was significantly impacted by the COVID-19 pandemic, during which approximately 43% of the affected participants missed consecutive doses of the exon-skipping therapy. Although a post hoc analysis excluding these individuals suggested a clinically meaningful delay in disease progression, the non-pre-specified nature of this analysis warrants cautious interpretation. The company also emphasized the totality of evidence, pointing to a significant increase in dystrophin protein expression and a consistent delay of approximately 5 months in the median time to functional decline across other trajectory-based post hoc analyses [47]. Following these results, close attention is now focused on the FDA’s decision regarding Sarepta’s subsequent sNDA submissions seeking to convert the drugs’ accelerated approvals to traditional approvals [48].

Additionally, a post-marketing long-term observational study, the phase 4 clinical trial NCT06606340 (EVOLVE), is currently underway and is expected to follow patients for up to five years [34].

## 4. Viltolarsen (Viltepso)

Viltolarsen developed by NS Pharma is an ASO designed to skip exon 53 and was granted FDA approval in 2020. Development proceeded after an optimal PMO sequence (NS-065/NCNP-01) for exon 53 skipping was identified *in vitro* [49]. Table 4 summarizes the main clinical trials related to viltolarsen.

**Table 4.** Major clinical trials of viltolarsen.

NCT number (Study name)	Phase	Study overview	Patient profile, dosing scheme	Key outcomes
NCT02081625 (NCNP/DMT01)	1	Open-label study to assess the safety, tolerability, efficacy and pharmacokinetics of viltolarsen	10 male DMD patients (ages 5–18) received 1.25, 5, or 20 mg/kg IV once weekly for 12 weeks.	Good safety profile. Dose-dependent exon skipping.
NCT02740972 (NS-065/NCNP-01–201)	2	Randomized study to assess the safety and tolerability, and efficacy of viltolarsen	16 boys with DMD (ages 4–10) received 40 or 80 mg/kg IV once weekly for 24 weeks (first 4 weeks were a double-blind study including placebo).	At 25 weeks, dystrophin expression was 5.7% (40 mg/kg group) and 5.9% (80 mg/kg group). Favourable 6MWD compared to external natural history cohort.
NCT03167255	2	Open-label extension of NCT02740972	Patients who completed NCT02740972 received viltolarsen for 192 weeks.	Good maintenance of motor function when compared with historical controls.
NCT04687020 (VILT-502)	4	Open-label extension of NCT03167255 to assess the long-term safety and efficacy of viltolarsen	Patients who completed NCT03167255 received 80 mg/kg viltolarsen for 120 months.	Study completion expected in 2032.
NCT04956289 (Galectic53, NS-065/NCNP-01–211)	2	Open-label study to assess the safety and tolerability, and efficacy of viltolarsen	20 DMD patients (including 10 non-ambulatory patients) aged 8 and older received 80 mg/kg IV once weekly for 48 weeks.	At 49 weeks, maintenance of upper limb function and lung function compared to external natural history cohort.
NCT04060199 (RACER53, NS-065/NCNP-01–301)	3	Randomized, double-blind, placebo-controlled study to assess the efficacy and safety of viltolarsen	77 boys with DMD (ages 4–7) received viltolarsen 80 mg/kg or placebo IV once weekly for 48 weeks.	At 48 weeks, the time to stand from supine velocity did not reach statistical significance.
NCT04768062 (RACER53-X, NS-065/NCNP-01–302)	3	Open-label extension of NCT04060199	Patients who completed NCT04060199 received 80 mg/kg for 96 weeks.	Ongoing; results pending (estimated study completion: Nov 2025)

Information based on ClinicalTrials.gov and announcement from the company as of May 2026. DMD, Duchenne muscular dystrophy; IV, intravenous; 6MWD, 6-minute walk distance.

NCT02081625 was a phase 1, open-label, dose-escalation clinical trial of viltolarsen [50]. Ten male patients aged 5 to 18 were divided into three dose groups (1.25, 5, and 20 mg/kg) and received weekly intravenous infusions for 12 weeks. Results confirmed a favourable safety profile and the ability to induce exon skipping. In Japan, a dose-setting study was conducted as an open-label phase 1/2 trial (JapicCTI-163291). Viltolarsen was administered at 40 mg/kg or 80 mg/kg weekly for 24 weeks. The 80 mg/kg dose demonstrated superior exon skipping and dystrophin expression, and no serious adverse events were observed [51,52].

NCT02740972 was a phase 2 clinical study conducted in North America to evaluate safety, tolerability, and intramuscular dystrophin protein expression [53]. Sixteen ambulatory boys aged 4 to 10 were assigned to either a 40 mg/kg group (*n* = 8) or an 80 mg/kg group (*n* = 8), receiving weekly infusions for 24 weeks. The trial consisted of a 4-week randomized double-blind period (for safety confirmation) followed by a 20-week open-label treatment period. After 20–24 weeks of treatment, significant dystrophin production was confirmed in both groups, reaching 5.7% of normal levels in the low-dose group and 5.9% in the high-dose group. Furthermore, functional improvements were noted when compared to an external natural history control (*n* = 65 untreated patients); the treated group showed a +28.9 m change in the 6MWT, whereas the control group showed a −65.3 m decline. The drug exhibited excellent tolerability, with no serious adverse events or cases requiring dose reduction or discontinuation. In the subsequent open-label extension study, NCT03167255, viltolarsen-treated patients were followed for up to 192 weeks. Findings indicated a favourable maintenance of motor function compared to historical controls [54,55]. These patients are scheduled for further follow-up for up to 10 years in NCT04687020 (VILT-502) [56].

NCT04956289 (Galactic53) was a phase 2 study involving 20 DMD patients, including 10 non-ambulatory patients aged 8 years or older [57]. Participants received 80 mg/kg of viltolarsen weekly for 48 weeks and were compared to a natural history cohort. At week 49, the treated group showed better pulmonary function, including a higher percent predicted forced vital capacity and higher peak cough flow, along with maintenance of upper limb function. This suggested that viltolarsen could be a viable therapeutic option for non-ambulatory as well as ambulatory patients.

The confirmatory phase 3 randomized, double-blind, placebo-controlled trial, NCT04060199 (RACER53), was conducted to verify the clinical benefit required for the FDA’s accelerated approval. Seventy-seven ambulatory DMD boys (aged 4–7) were randomized to either 80 mg/kg of viltolarsen or a placebo for 48 weeks. According to a press release, the primary endpoint, time to stand from supine velocity at week 48, failed to achieve statistical significance compared to the placebo group, despite demonstrating a favourable upward trend in the viltolarsen arm [58]. The open-label extension study, NCT04768062 (RACER53-X), is currently ongoing to track changes in motor function during up to 96 weeks of viltolarsen administration [59].

## 5. Casimersen (Amondys 45)

Casimersen developed by Sarepta Therapeutics is an ASO designed to induce the skipping of exon 45 and received FDA approval in 2021. This agent is applicable to approximately 13.1–15.1% of DMD patients with deletion mutations, representing 9.0–9.1% of the total DMD population [15,16]. Table 5 summarizes the main clinical trials related to casimersen.

**Table 5.** Major clinical trials of casimersen.

NCT number (Study name)	Phase	Study overview	Patient profile, dosing scheme	Key outcomes
NCT02530905	1	Randomized, double-blind, placebo-controlled study to assess the safety, tolerability and pharmacokinetics of casimersen	12 male DMD patients (ages 7–21) with limited or no ambulation received 4, 10, 20, 30 mg/kg via dose escalation IV once weekly for 12 weeks. All participants received casimersen 30 mg/kg up to 144 weeks in the subsequent open-label extension.	Showed good tolerability; no serious adverse events.
NCT02500381 (ESSENCE, 4045–301)	3	Randomized, double-blind, placebo-controlled study to evaluate the efficacy and safety of casimersen (and golodirsen)	228 boys with DMD (ages 6–13) received 30 mg/kg or placebo IV once weekly for 96 weeks. All participants received active treatment for 48 weeks in the subsequent open-label extension.	At 48 weeks, increase in exon skipping and dystrophin expression (1.74%) from baseline. At 96 weeks, the change from baseline in the 4-step ascend velocity did not reach statistical significance.
NCT06606340 (EVOLVE, 4658–403)	4	Long-term observational study of casimersen	30 mg/kg IV once weekly (target enrolment: 300 patients).	Study completion expected in 2033.

Information based on ClinicalTrials.gov and announcement from the company as of May 2026. DMD, Duchenne muscular dystrophy; IV, intravenous.

NCT02530905 was a phase 1 clinical trial that evaluated the safety, tolerability, and pharmacokinetics of casimersen. While the study design was similar to the phase 1/2 trial for golodirsen, it specifically focused on 12 DMD patients aged 7 to 21 with limited or no ambulation [60]. Participants received weekly intravenous infusions of SRP-4045 (casimersen) with dose escalation (4, 10, 20, and 30 mg/kg) or placebo during a 12-week randomized, double-blind period, followed by an open-label extension for up to 132 weeks. Casimersen demonstrated a favourable safety profile with no serious adverse events, and continuous administration at 30 mg/kg did not result in plasma accumulation. Notably, muscle biopsy evaluations were not performed in this specific trial.

The clinical efficacy of casimersen was evaluated through the NCT02500381 (ESSENCE trial), a larger-scale phase 3, double-blind, randomized, placebo-controlled study [45]. Ambulatory DMD boys aged 7–13 were randomized 2:1 to receive either 30 mg/kg of casimersen or a placebo via weekly intravenous infusion for 96 weeks. Notably, an interim analysis of this trial at week 48 served as the primary basis for the FDA’s accelerated approval of casimersen in 2021; the analysis demonstrated biomarker improvement – with all 27 patients in the casimersen group exhibiting exon 45 skipping and an increase in dystrophin protein levels from 0.93% to 1.74% [61,62]. However, this surrogate benefit has not yet translated into established clinical efficacy. Specifically, in early 2026, Sarepta Therapeutics released top-line results stating that the primary endpoint, change from baseline in the 4-Step ascend velocity at week 96, did not reach statistical significance [46]. Although the company has since presented functional outcome data from post hoc analyses, intense attention is now focused on how the FDA will proceed regarding the conversion to full approval in light of these results (see also the section on golodirsen).

As part of post-marketing surveillance, the phase 4 clinical trial NCT06606340 (EVOLVE) is currently underway, which will track long-term safety and efficacy data for up to five years [34].

## 6. Development of next-generation ASOs

As described above, while the development of four FDA-approved ASO drugs has demonstrated the feasibility of exon skipping strategies, human clinical trials have yet to demonstrate the level of efficacy, such as dystrophin restoration and improvement of motor function, that was anticipated based on animal studies. To overcome this limited efficacy, the development of next-generation ASOs is being actively pursued (Table 6). In particular, strategies utilizing bioconjugation technology to improve delivery efficiency are being aggressively explored. Here, we first introduce two strategies focused on improving delivery efficiency and muscle-specific targeting that have already advanced to the clinical trial stage, followed by an introduction to other promising technologies and therapeutic concepts.

**Table 6.** Comparison of FDA-approved exon skipping ASOs for DMD and representative next-generation ASOs currently in development or previously under development.

Drug name	Company	Platform	Target exon	Clinical status (as of May 2026)
Eteplirsen	Sarepta Therapeutics	Naked PMO	51	FDA-approved
Golodirsen	Sarepta Therapeutics	Naked PMO	53	FDA-approved
Viltolarsen	NS Pharma	Naked PMO	53	FDA-approved
Casimersen	Sarepta Therapeutics	Naked PMO	45	FDA-approved
Vesleteplirsen (SRP-5051)	Sarepta Therapeutics	PPMO	51	Development discontinued
PGN-EDO51	PepGen	PPMO	51	Development discontinued
ENTR-601–44	Entrada Therapeutics	PPMO	44	Phase 1/2 NCT07037862 (ELEVATE-44): ongoing
Delpacibart zotadirsen (AOC-1044)	Avidity Biosciences	AOC: anti – TfR1 antibody + PMO	44	Phase 1/2 NCT06244082 (EXPLORE44OLE): OLE ongoing
Z-rostudirsen (DYNE-251)	Dyne Therapeutics	AOC: anti – TfR1 antibody + PMO	51	Phase 1/2 NCT05524883 (DELIVER): OLE ongoing; Phase 3 NCT07608432 (FORZETTO): ongoing
Suvodirsen	Wave Life Sciences	Steropure ASO with PS backbone	51	Development discontinued
WVE-N531	Wave Life Sciences	Stereoselective PS and PN backbone with sugar modification	53	Phase 1/2 NCT04906460 (FORWARD-53): ongoing
SQY51	SQY Therapeutics	Tricyclo-DNA	51	Phase 1/2 NCT05753462 (AVANCE1): awaiting analysis results
Brogidirsen (NS-089/NCNP-02)	NS Pharma	Dual-targeting PMO	44	Phase 2 NCT05996003: ongoing

ASO, antisense oligonucleotide; DMD, Duchenne muscular dystrophy; PMO, Phosphorodiamidate morpholino oligomer; PPMO, Peptide-conjugated PMO; AOC, Antibody-Oligonucleotide Conjugate; TfR1, Transferrin receptor 1; OLE, open-label extension; PS, phosphorothioate; PN, phosphoryl guanidine.

The first strategy involves peptide-conjugated PMOs (PPMOs), which utilize cell-penetrating peptides (CPPs). vesleteplirsen (SRP-5051), developed by Sarepta Therapeutics, is a next-generation ASO featuring a CPP conjugated to eteplirsen. In the phase 2 MOMENTUM trial (NCT04004065), it demonstrated exon 51 skipping efficiency and dystrophin restoration levels that surpassed its predecessor [48,63]. However, hypomagnesaemia – indicative of renal tubule damage – was observed in numerous cases and persisted in some patients even after treatment discontinuation, ultimately leading to the termination of its development [64]. While non-clinical studies had previously flagged renal toxicity as a potential hazard of CPP-conjugated platforms [65,66], the emergence of persistent hypomagnesaemia in humans underscored a critical hurdle for translating PPMOs into clinical practice. Similarly, PGN-EDO51, a PPMO developed by PepGen for exon 51 skipping, underwent evaluation in the phase 2 CONNECT1-EDO51 trial (NCT06079736). Although not as pronounced as with vesleteplirsen, asymptomatic hypomagnesaemia was also observed in some participants in the PGN-EDO51 high-dose cohorts [67]. Interestingly, while PGE-EDO51 showed high levels of exon skipping and dystrophin restoration in preclinical trials [68,69]、its performance in patients fell short; dystrophin restoration remained at a low level, leading to the subsequent announcement that further development of the agent would be discontinued [70,71]. This highlights the substantial gap that still exists between preclinical efficacy and real-world clinical outcomes. Currently, Entrada Therapeutics is developing the exon 44 skipping PPMO drug ENTR-601–44 utilizing its Endosomal Escape Vehicle (EEV) platform. The candidate is currently being evaluated in the phase 1/2 ELEVATE-44 study (NCT07037862) [72]. According to the recently announced top-line results, an interim analysis of the first cohort reported that markers of kidney function, including serum magnesium levels, remained within the normal range [73]. Longer-term data and results from the second cohort receiving higher doses are being closely monitored.

The second strategy involves approaches using cell surface-specific antibodies. This strategy optimizes muscle delivery by conjugating PMOs with antibodies targeting proteins highly expressed in skeletal and cardiac muscle, such as the transferrin receptor 1 (TfR1) [74]. AOC-1044, an antibody-oligonucleotide conjugate (AOC) being developed by Avidity Biosciences, utilizes a monoclonal antibody targeting TfR1 to deliver a PMO designed for exon 44 skipping. It demonstrated superior tissue penetration in mouse models [75]. In the phase 1/2 EXPLORE44 trial (NCT05670730), it showed a favourable safety profile and high delivery efficiency to muscle tissue [76]. An open-label extension study, EXPLORE44OLE (NCT06244082), to verify long-term safety is currently ongoing [77]. Additionally, Dyne Therapeutics is developing z-rostudirsen (DYNE-251), a next-generation agent designed for exon 51 skipping conjugated to an antibody Fc fragment that binds to TfR1. In the phase 1/2 DELIVER trial (NCT05524883), top-line results released in late 2025 reported a significant increase in muscle-content adjusted dystrophin to 5.46% at 6 months, which served as the primary endpoint. Additionally, post hoc analyses of these top-line data suggested preliminary improvements in motor and respiratory function [78,79]. Reflecting its therapeutic potential, the FDA has granted Breakthrough Therapy Designation to z-rostudirsen. The company subsequently submitted a Biologics License Application for accelerated approval in May 2026. Concurrently, Dyne Therapeutics initiated the global confirmatory phase 3 trial (NCT07608432) in May 2026, with a design aligned with FDA guidelines [79,80]. Given its potential for once-monthly dosing, it may overcome many of the shortcomings of conventional agents, although its long-term clinical efficacy awaits validation.

Notably, animal studies have confirmed that these approaches achieve myocardial uptake – a feat that was difficult to accomplish with conventional PMOs alone – suggesting potential therapeutic efficacy in the heart as well (although peer-reviewed publications validating the cardiac effects of SRP-5051, PGN-EDO51, and DYNE-251 remain unavailable to date) [75,81]. While current clinical trials do not directly evaluate or measure cardiac efficacy as an endpoint, these approaches are expected to eventually have a major impact on preventing or delaying the progression of DMD cardiomyopathy, which remains one of the primary causes of death in DMD patients [82]. Similarly, development is also underway for blood-brain barrier-crossing ASO platform technologies aimed at targeting the central nervous system, which represents another crucial therapeutic target tissue.

Apart from bioconjugation, development efforts focusing on the chemical modification of ASOs are also underway [83]. Wave Life Sciences developed suvodirsen, an exon 51 skipping drug, by utilizing a stereopure backbone linkage technology that fully controls the Rp/Sp stereochemistry of phosphorothioate linkages. Despite promising results in preclinical studies, no increase in dystrophin protein expression was observed in phase 1 (NCT03508947) [84]. Consequently, the phase 2/3 DYSTANCE 51 trial (NCT03907072) was discontinued, leading to the termination of its development [85]. Subsequently, the company advanced the development of WVE-N531, an exon 53 skipping drug, by leveraging a platform that combines chemistry modifications, specifically a mixture of stereoselective phosphorothioate and phosphoryl guanidine backbone linkages, with sugar modifications. The phase 1/2 FORWARD-53 trial (NCT04906460) is currently ongoing. Interim analysis reports from the company have demonstrated significant restoration of muscle content adjusted dystrophin as measured by Western blot – reaching 9.0% at week 24 and 6.4% at week 48— and improvements in certain functional assessments, and development strategies are now moving forward with a view towards accelerated approval [86,87]. In addition, as an ASO employing an entirely different chemical structure, SQY Therapeutics is developing SQY51, an exon 51 skipping drug that utilizes tricyclo-DNA. It is expected to distribute not only to skeletal muscle but also to cardiac muscle and the central nervous system, and the analysis results of the phase 1/2 AVANCE1 trial (NCT05753462) are currently awaited [88].

In addition to improving efficacy, expanding treatment access is a critical priority. While development has prioritized mutations covering larger patient populations (e.g. exons 51, 53, and 45), there is a need for a framework to support the development for patients with rarer mutations by applying accumulated expertise. Currently, drugs targeting exon 44 skipping (brogidirsen, AOC-1044, ENTR-601–44) are nearing practical application [89,90]. Furthermore, there is an urgent need for treatments for patients with exon 8 or 9 deletions, who are currently ineligible for micro-dystrophin therapy. Research into multi-exon skipping (e.g. skipping 45–55, 6–8, or 3–7) is also progressing as a means of expanding indications [91–93]. However, the use of ASO ‘cocktails’ increases the total dosage, potentially raising the risk of adverse events and sequence-independent off-target effects, necessitating cautious validation.

## 7. Future directions

The emergence of four exon-skipping PMO agents has transformed the treatment of dystrophin intervention from a theoretical concept into a clinical reality, dramatically shifting the DMD treatment paradigm over the past decade. The approval of gene replacement therapies using micro-dystrophin has also exerted a decisive influence on the architecture of DMD therapeutic strategies [11,94]. While these therapies possess inherent limitations – namely, that their therapeutic concept relies on restoring partially functional dystrophin to mimic a milder BMD phenotype, and that they are initiated at a stage where a certain degree of irreversible muscle damage has already occurred – they represent a major departure from conventional treatments as a means of directly addressing dystrophin deficiency. The process spanning from serial drug development to FDA approval, along with post-marketing trends, has established a pioneering and invaluable platform for intractable hereditary rare diseases. Concurrently, however, it has brought various challenges and unmet medical needs into sharp focus.

The limited efficacy of ASOs and the lack of evidence regarding clinical outcomes represent major challenges in this field. Because these FDA approvals were primarily granted based on biomarker restoration, controversies persist over approving these drugs when definitive clinical benefits remain unproven. Importantly, it must be acknowledged that absolute dystrophin percentages do not inherently reflect therapeutic efficacy; the clinical outcome is tightly governed by the quality of the restored protein, including its proper cellular localization, molecular stability, and tissue distribution. Therefore, biomarker restoration alone provides an incomplete picture of biological functionality. Although designing clinical trials for rare, mutation-specific diseases is inherently challenging, trial designs have become increasingly sophisticated – refining patient selection, establishing appropriate control groups, and identifying clinically meaningful endpoints. Nevertheless, no double-blind, placebo-controlled trial evaluating these FDA-approved agents has yet demonstrated superiority in functional primary endpoints. While post hoc analyses or comparisons with external cohorts provide valuable insights, their strength as clinical evidence is inherently limited. Crucially, as non-pharmacological therapies and mutation-independent DMD treatments advance, comparisons with historical or external cohorts will become even more difficult to interpret. Currently, the FDA is in a precarious position regarding the regulatory status of these drugs. This regulatory decision is deeply intertwined with multiple, competing factors: the fatal nature of DMD, the safety profiles of the drugs, astronomical development costs, and the earnest expectations of patients and their families. Ultimately, a more sustainable future depends on the emergence of next-generation therapeutics that can demonstrate clear functional benefits in rigorous, placebo-controlled trials, thereby convincing both the medical community and society at large.

Furthermore, challenges regarding the real-world utilization of these agents after FDA approval also remain. First, none of the four exon-skipping drugs have been approved in Europe, and only viltolarsen has received approval in Japan, highlighting a stark disparity in current global access [51,83]. In addition, the financial burden imposed on patients’ families and society by these expensive drugs, as well as the burden of continuous, weekly intravenous administration, is substantial. Reports evaluating the real-world use of ASO agents have also pointed out that real-world patients are significantly older and present with more advanced disease progression compared to those enrolled in clinical trials, suggesting that the clinical efficacy may be even more limited in practice [95]. Moving forward, it will be important to conduct research and development as well as regulatory reviews that anticipate and account for these real-world disparities and limitations.

The trajectory of FDA-approved ASO development in DMD symbolizes a critical milestone in precision medicine. However, because current commercial strategies focus predominantly on hotspot mutations, patients with much rarer mutations are left without these therapeutic options. To truly embody the ethos of precision medicine, it is necessary to expand our horizon towards ‘N-of-1’ therapies; however, whether the feasibility of N-of-1 therapies utilizing exon-skipping ASOs can be realized in DMD remains an open question. As highlighted by a recent use-case analysis applying the N-of-1 development roadmap established by the International Rare Diseases Research Consortium (IRDiRC) to DMD, the core issue once again lies in the fact that the clinical efficacy of ASOs has not been fully demonstrated in clinical trials, leaving the exact extent to which they delay disease progression unknown [96,97]. Consequently, conducting a definitive risk-benefit assessment for individual patients is challenging, and determining which endpoints are appropriate for tracking disease progression remains elusive. Furthermore, compared to lower-dose local administration for central nervous system or ocular diseases, the costs for manufacturing and toxicology testing escalate drastically due to the high-dose systemic administration required to target muscle tissue. In this way, accelerating the development of next-generation therapeutics is also indispensable from the perspective of precision medicine.

Backed by extensive research and a track record of FDA-approved drugs, the development and clinical trial platforms for ASOs offer immense value for future drug development. Even as alternative approaches advance, including non-dystrophin-targeted therapies, non-pharmacological treatments, and other dystrophin-targeting modalities like gene editing, ASO therapeutics remain a highly viable and promising strategy. It is highly expected that in the near future, optimized molecularly targeted therapies will become accessible to all patients with DMD.

## Data Availability

Data sharing is not applicable to this article as no new data were created or analysed in this study.
